# End-Stage Renal Disease-Related Accelerated Immune Senescence: Is Rejuvenation of the Immune System a Therapeutic Goal?

**DOI:** 10.3389/fmed.2021.720402

**Published:** 2021-09-03

**Authors:** Didier Ducloux, Mathieu Legendre, Jamal Bamoulid, Philippe Saas, Cécile Courivaud, Thomas Crepin

**Affiliations:** ^1^Inserm, UMR1098, Federation Hospitalo-Universitaire INCREASE, Besançon, France; ^2^University Bourgogne Franche-Comté, Faculté de Médecine et de Pharmacie, LabEx LipSTIC, Besançon, France; ^3^Structure Fédérative de Recherche, SFR FED4234, Besançon, France; ^4^CHU Besançon, Department of Nephrology, Dialysis, and Renal Transplantation, Besançon, France; ^5^EFS Bourgogne Franche-Comté, Plateforme de Biomonitoring, CIC 1431/UMR1098, Besançon, France

**Keywords:** immune senescence, thymus, inflammaging, end-stage renal disease, kidney transplantation

## Abstract

End-stage renal disease (ESRD) patients exhibit clinical features of premature ageing, including frailty, cardiovascular disease, and muscle wasting. Accelerated ageing also concerns the immune system. Patients with ESRD have both immune senescence and chronic inflammation that are resumed in the so-called inflammaging syndrome. Immune senescence is particularly characterised by premature loss of thymic function that is associated with hyporesponsiveness to vaccines, susceptibility to infections, and death. ESRD-related chronic inflammation has multiple causes and participates to accelerated cardiovascular disease. Although, both characterisation of immune senescence and its consequences are relatively well-known, mechanisms are more uncertain. However, prevention of immune senescence/inflammation or/and rejuvenation of the immune system are major goal to ameliorate clinical outcomes of ESRD patients.

## Introduction

Patients with end-stage renal disease (ESRD) are especially prone to infection (1). Furthermore, concordant data also report that immune responses against vaccines are considerably reduced in this population (2). Concomitantly, ESRD patients exhibit aseptic low-grade inflammation (3, 4). These clinical features are close to those observed in elderly and suggest that inflammaging associating both premature senescence of the immune system and inflammation is a key part of the ESRD-related immune phenotype. What is more, some convincing studies established evidences of accelerated immune senescence in chronic kidney disease and dialysis patients compared to the general population (5–7).

In this review, we analyze recent knowledge on ESRD-associated accelerated immune ageing with a special focus on thymus involution. In addition, we speculate on therapeutic tools likely to prevent or reverse these immune alterations.

## Immune Senescence in Ageing

The term immune senescence clusters all the changes that occur in the immune system during ageing. Although this process mainly affects T lymphocytes, all aspects of innate and adaptive immunity are concerned. Recently, immune ageing has been suggested to be more appropriate to design all immune changes associated with ageing. Indeed, the ageing of the immune system is a more general concept including two different processes. The first one is what specifically refers to immune senescence, which is mainly linked to age-dependent thymic involution leading to reduced immune repertoire diversity and compounded oligo-clonal increase in memory immune cells. Sensitivity to infections, reduced vaccine immunity, and defect in tumour clearance observed in elderly are thought to be at least in part linked to these immune alterations. Immune senescence in T cells is sometimes called cellular exhaustion even if the two phenomenon are not exactly identical. Exhausted T cells are defined by the loss of CD28 and the concomitant expression of Tim-3 and PD-1 (8). The second characteristics of aged immunity is inflammaging. Old age is associated with low-grade systemic inflammation. Chronic innate immune activation, pro-inflammatory cytokine profile secretion, and age-induced accumulation of self-reactive T cells contribute to age-related inflammation. Inflammaging is supposed to explain some degenerative disease associated with ageing. The term “Inflammaging” is frequently proposed to include these two aspects.

### Immune Senescence: A Pivotal Role of Thymic Involution

T cell immune senescence is mainly linked to physiologic thymic involution. The thymus mainly serves to the development of a large but self-tolerant T cell repertoire. Briefly, multipotent hematopoietic stem cells (HSC) differentiate into common lymphoid or myeloid progenitors. T lymphoids precursors go to the thymus where they undergo several stages of maturation resulting in the formation of naive T lymphocytes called recent thymic emigrant (RTE) (9). These cells present a diversified polyclonal T cell receptor (TCR). Central tolerance occurs in the thymus *via* two mechanisms. The first one is thymocyte negative selection. This step consists in deletion of most of self-auto-reactive T cells *via* apoptosis (9). The second concerns the generation of CD4 single positive FoxP3+ regulatory T cells, which can eliminate auto-reactive T cells having escaped to negative selection (9).

The ability to generate RTE in the thymus declines with age. Thymic involution consists in reduction of both thymic size and thymocyte number and reorganisation of thymic ultrastructure. Soon after birth, functional tissue begins to be substituted by fat (10). After 50 years, there is almost no output of naïve T cells. The frequency of naïve T cells greatly diminishes both in periphery and in lymphoid organ, especially for CD8+ T cells. Nevertheless, homeostatic proliferation of previously generated naïve T cells enables to maintain a broad and diverse pool of naïve T cells, especially for CD4+ T cells. Continuous involution of the thymus with age finally causes a decrease in the thymic output of naïve T cells and subsequently a reduction of the peripheral TCR repertoire.

Two non-exclusive mechanisms account for thymus involution. The first one is mainly based on a reduced production of HSC. Indeed, self-renewal of HSC diminishes with age and tends to favour myeloid lineage (11, 12). Reduction in HSC production and switch toward myeloid lineage would both contribute to decrease the output of common lymphoid progenitors (13, 14). In addition, aged hematopoietic stem cells have less lymphoid differentiation potential (15). The second one depends on age-related reduction in stromal niches of the bone marrow () and thymus (16, 17). Recent studies mainly plead for the latter hypothesis. Stroma cells in the thymus are mainly thymic epithelial cells (TECs) (18). Convincing data show that age-associated thymic involution is dependent on TEC transcription factors involved in TEC homeostasis, such as Forkhead box N1 (19). Indeed, FOXN1 is essential for thymus development and thymocyte formation (19). A null mutation in the FOXN1 gene defines the “null mice” which phenotype is characterised, amongst others, by the absence of thymus and T cells (20). Reduction in thymic FOXN1 expression is observed as one the first step of thymic involution in aged individuals (21). Conditional KO mice studies have considerably explained the causal role of FOXN1 in thymus involution. LoxP-floxed-FoxN1 mouse with the ubiquitous CreER(T) transgene have a low dose of spontaneous activation and exhibit progressive loss of FOXN1 (22). Progressive loss of FOXN1 is associated with accelerated thymic involution (22). Finally, intra-thymic supplementation in FOXN1-cDNA partially reverses thymic involution and restores peripheral CD4+ T cell population (22).

The decrease in T cell production is compensated by homeostatic expansion of existing peripheral T cells occurs. This leads to an increased proportion of memory T cells and reduction in the diversity of TCR repertoire (23). Accumulation of memory T cells is mainly due to life-long exposure to chronic antigen stimulation by pathogens. The most important is cytomegalovirus. However, expansion of CD8+ T cells is only observed in CMV-exposed old patients (24).

### Inflammaging

Somatic cellular senescence is defined by the permanent arrest of cell cycle accompanied by lack of proliferation, expression of anti-proliferative markers, and shortening of telomeres (25). This biological process is likely to be protective against cancer transformation (26).

Accumulation of somatic senescent cells contribute on one hand to organ dysfunction, and on the other hand to inflammation through induction of somatic cell senescence-associated secretory phenotype (SASP). Immune senescence favours increased production of SASP (27) due to decreased chemotaxis of immune cells toward somatic senescent cells and reduced phagocytosis by neutrophils and macrophages (28–31).

Many other mechanisms contribute to inflammation in ageing. Chronic viral infections, especially with CMV, induce low level of cytokines production (32).

Moreover, involution of the thymus is accompanied by a decrease ability to negatively select self-auto-reactive T cells, which explain propension to certain autoimmune diseases in the elderly (33). Paradoxically, peripheral Treg cells accumulate during ageing. Thomas et al. (34) developed a mock-self-antigen chimaera mouse model, in which membrane-bound ovalbumin transgenic mice, carrying a FOXN1-floxed gene for induction of conditional thymic atrophy, received ovalbumin-specific T cell receptor transgenic progenitor cells. The authors showed that a decreased number of ovalbumin-specific tTreg and pTreg, but not polyclonal Treg cells in chimeric mice with thymus atrophy. The ovalbumin-specific pTreg had less suppressive activity and a lower expression of FoxP3. This suggest that although generation of polyclonal pan-Treg is not affected by thymus involution, certain specific Treg clones may have aberrant agonist selection contributing to age-related chronic inflammation.

Thus, immune ageing is characterised by both immune deficiency (immune senescence driven by thymus involution) and inflammation leading to the concept of inflammaging.

## Immune Ageing in End-Stage Renal Disease

Chronic kidney disease phenotype is very similar to premature ageing. Frailty, osteoporosis, muscle wasting, and cardiovascular disease occur at younger age in CKD patients. Many factors such as oxidative stress, accumulation of uremic toxins, and inflammation are supposed to contribute to accelerated ageing (35). The immune system undergoes a similar premature ageing. Indeed, peripheral blood mononuclear cell relative telomere length is shorter in CKD patients as compared to healthy individuals (5). Furthermore, ESRD patients frequently exhibit T cell lymphopenia (6) and concomitantly have both a marked susceptibility for infections and a decreased response to vaccines suggesting a T cell immune defect (7). Finally, ESRD patients exhibit a low-grade inflammation status (36). This association is typical of the “inflammaging” state observed in elderly.

Premature thymic involution is a key component of ESRD-associated immune senescence. Others and we reported that thymic output decreased with progression of CKD. Thymic output is comparable between 40-year-old uremic patients and 80 year-old non-uremic patients (5). Our group recently reported that, in ESRD patients, low thymic output was predictive of severe infections (5). The decrease in RTE could be the result of a reduction in the thymic output of naïve T cells and/or of a reduction in homeostatic proliferation. Premature loss of thymic function is likely to explain the decrease in naïve T cells in young patients with ESRD. Indeed, decreased CD4 naïve T cells percentage is also observed in paediatric CKD patients (37). Moreover, concordant data in animals suggest that acute renal failure accelerates thymus involution (38, 39).

However, there are few data documenting potential causes for premature thymic involution during chronic kidney disease. Chronic inflammation is likely to markedly contribute to immune ageing. Of note, a recent study shows that CRP levels inversely correlates with naïve T cells in haemodialysis patients suggesting either that inflammation and immune senescence evolve in parallel or that one is driving the other one (40). Activation of innate immunity, characterised by monocyte activation and overproduction of inflammatory cytokines such as Il-6, is a key feature of the CKD immune system (4, 41, 42). Thus, Jurk et al. (43) reported that knockout of the nfkb1 subunit of the transcription factor NF-κB induces chronic low-grade inflammation that leads to premature ageing in mice. Treating reversible source of inflammation is obviously a goal in CKD patients and such strategy may reduce premature ageing.

**Figure 1:**
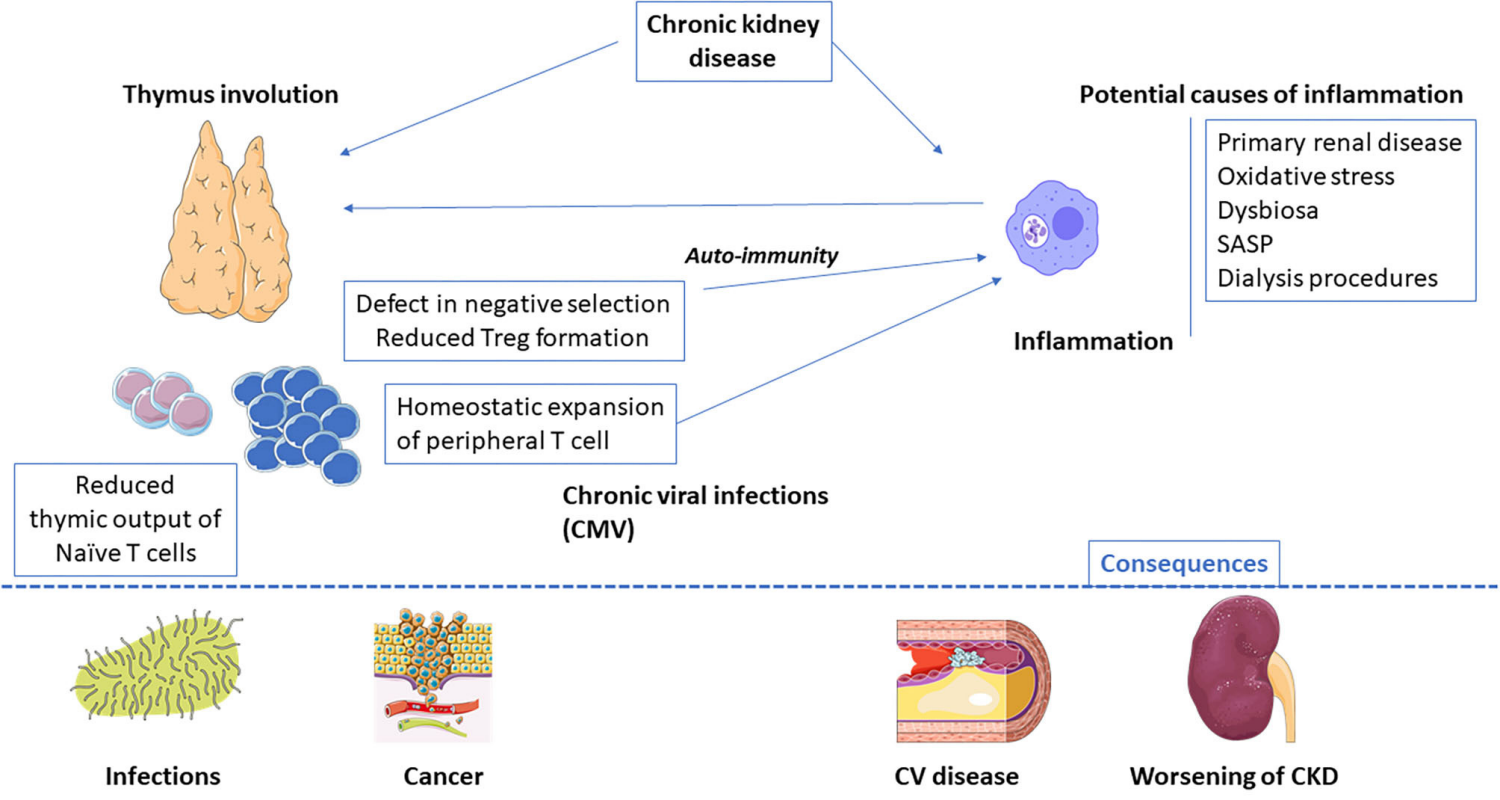
End-stage renal disease-related inflammaging: main causes and potential consequences. CMV, Cytomegalovirus; SASP, senescence-associated secretory phenotype.

Main mechanisms of premature immune ageing are summarised in Figure 1.

## Immune Rejuvenation: Facts and Perspectives in CKD

**Figure 2:**
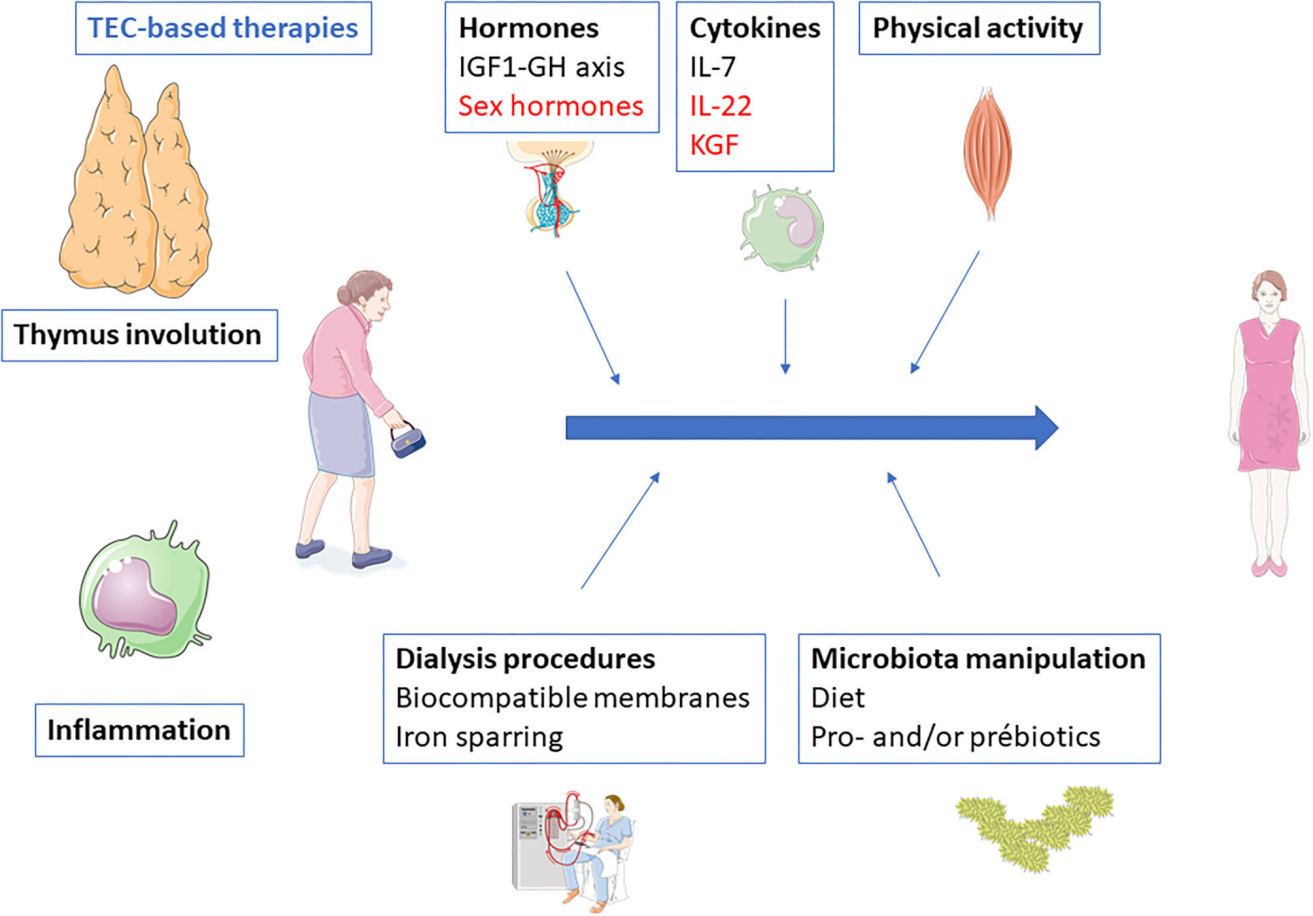
Potential therapeutic interventions to prevent or reverse end-stage renal disease-related inflammaging. Black, interventions with evidences; Blue, interventions without clinical evidence but with strong background and potentiality; Red, interventions with evidences of inefficacy.

Immune senescence has deleterious consequences. Susceptibility to infection, premature cardiovascular disease, and increased cancer incidence are some of the most frequent and serious. A number of measures, from the simplest to the more complex, may be susceptible to reverse immune senescence, especially premature thymic involution (Figure 2).

## Physical Activity

The impact of physical activity in maintaining thymic activity must not be neglected. It is one of the rare therapeutic strategies with consistent results in both animal and human studies (44).

In an immunological ageing mouse model, 4 weeks of free-wheel running increased naïve T lymphocytes and reduced effector ratio of cytotoxic T lymphocytes (45). Concordant data also exist in humans. Comparing adults (55–79 years) who had intensive sportive practise (cycling), age-matched adults and young sedentary adults, Duggal et al. (46) observed increased frequency of naïve T cells and RTE in cyclists. Sportsmen had also higher levels of IL-7 and lower levels of IL-6. By contrast, CD28-CD57+CD8+ T cell frequencies did not differ between the three groups. Even more powerful are the evidence that sustained physical activity in elderly improve immune responses against influenza vaccine (47, 48).

Skeletal muscles express and secrete different cytokines, also called myokines. Among them, IL-7 and IL-15 are released during exercise (49, 50). IL-6 is also released by muscle during exercise. Nevertheless, whereas IL-6 secreted through NF-KB signalling is pro-inflammatory, IL-6 produced by muscles is dependent on JUN N-terminal kinase and activator protein 1 signalling and exhibits anti-inflammatory properties (51, 52). Proof of concept is supported by experiments showing that both exercise and IL-6 infusion suppress inflammation induced by endotoxin injection (53). Modulation of cytokine secretion by muscles during exercise are likely to explain the link between physical activity and thymopoiesis.

Physical activity is often reduced in CKD patients. Sedentary life, socio-economics conditions, comorbidities, and uremia-related asthenia contribute to the reduced physical activity. Although a large number of studies reported the beneficial effects of exercise in CKD patients, no data are available concerning the potential consequences on immune status. However, other benefits of physical exercise in ESRD patients have been largely reported and physical rehabilitation programs should be encouraged in these patients. Further studies should analyze whether physical activity may at least in part reverse or prevent thymic involution and inflammation.

## Hormones

Many hormonal pathways play a role in thymic physiology. However, most of them are impaired during chronic renal failure.

### IGF-1-GH Pathway

The IGF-1–GH pathway interferes with many aspects of thymus biology. TECs express GH receptors (54) and IGF is expressed in the thymus (55). Growth hormone supplementation increases thymic cytokine production and T cell progenitor recruitment into the thymus and can reverse thymic involution (56–58). Hansen et al. (59) reported that treatment with rhGH increased thymus size, T cell receptor excision circles (TREC) frequency, and total TREC content in CD4 T cells in HIV-infected patients. GH withdrawal in patients receiving GH treatment is followed by decreased thymic output and intra-thymic T cell proliferation (60).

The IGF-1-GH axis is profoundly altered in dialysis patients. ESRF patients have increased GH secretion, but normal IGF-1 concentrations, indicating GH resistance (61, 62). The resistant state is related to alterations at several levels of GH/IGF-1 axis, GH signalling, and IGF-1 action (63, 64). Several studies reported that GH administration might increase IGF-1 levels in dialysis patients as in healthy subjects (65). Moreover, large studies confirmed the safety of long-term administration of GH in dialysis patients (66).

All these data suggest that GH may be a therapeutic hope to reverse thymopoiesis defect in ESRD patients.

### Sex Hormones

The effects of sex hormones on thymus are well-known. A number of studies demonstrated that sex steroid ablation delay or reverse thymus involution in both animals and humans (67, 68). Sex steroids inhibit TEC expression of Notch ligand Delta-like 4 that promotes T cell differentiation and development (69).

Surgical castration is obviously not a therapeutic option in humans, but LHRH analogues use is also associated with thymic rejuvenation (70). Leuprolide desensitises LHRH receptors and reduce the release of LH and FSH. Goldberg et al. (71) showed that Leuprolide enhances T cell reconstitution following allogeneic bone marrow transplantation in mice. Similar data have been obtained in non-human primates (72). Leuprolide induces thymic rejuvenation in aged male baboons (72). Castration by Leuprolide is reversible and, due to the use of LHRH agonists in a variety of human diseases, safety, pharmacokinetics and efficacy are well-known.

Nevertheless, some studies also suggest that castration-induced thymic rejuvenation is only transient and potentially hazardous. Indeed, sex hormones deprivation favours self-reactivity (73, 74). Concordant with this concern, castration decreased CD4+CD25+ Treg and increased natural (NK) cells in humans (75). Moreover, androgens increase autoimmune regulator (AIRE) expression in mTEC and therefore enhance negative thymocyte selection while estrogens have opposite effects (76).

Despite some former results, the use of chemical castration to enhance thymic rejuvenation is consequently not a safe option.

## Cytokines

### IL-7

IL-7 is produced by both thymic stromal cells and bone marrow. IL-7 mediates lymphopoiesis of both T and B cells, and in the thymus, promotes proliferation, differentiation, and survival of thymocytes (77). IL-7 signals through its receptor IL-7R (78). Loss of function mutations in IL-7R leads to severe combined immunodeficiency (SCID) (78).

Administration of IL-7 in mice expand both naïve and memory CD4 and CD8 peripheral T cells (79).

RhIL-7 has been used in different clinical settings and constantly leads to increase circulating T cell populations, with more specific expansion of RTE, naïve T cells and central memory T cells (80–83). TCR repertoire diversification is also observed in rhIL-7 treated patients (84). The increase in both CD4+ and CD8+ T cell remain for months after the end of treatment by rhIL-7 (85).

IL-7 concentrations have been found to be elevated in CKD (86) suggesting a possible relative resistance to this cytokine. Nevertheless, to date, there is no study assessing the effects of rhIL-7 in lymphopenic CKD patients. Our group recently begun a phase II study (INDIA Study NCT…) using rhIL-7 to reverse thymic involution in ESRD patients on dialysis.

### IL-22

Interleukin-22, also called IL-10-related T cell-derived inducible factor (IL-TIF) (87), is a member of the IL-10 family, including IL-19, IL-20, IL-24, IL-26, IL-28, and IL-29. IL-22R1 determines the cellular sensitivity toward IL-22. This receptor is restricted to specific cell types and is absent on immune cells (88). Il-22 interacts with IL-2R on the surface of TEC and allows both survival and proliferation of thymocytes.

IL-22 administration to mice having received total body irradiation increases both thymocytes and TEC recovery (89). Similar observations have been done after murine allogeneic hematopoietic cell transplant (90). IL-22 increases the number of TEC *via* a stat3-dependent signalling (91). More recently, it was shown that, after allogeneic hematopoietic transplantation, plasma IL-22 levels positively correlated with blood TREC levels (92).

Limitations in the therapeutic use of rhIL-22 are based on its dual effects, which strictly depend on the context. The pro-regenerative effects of IL-22 could be counterbalanced by its inflammatory and tumorigenic properties.

### Keratinocyte Growth Factor

KGF belongs to the fibroblast growth factor family. This cytokine is involved in epithelial cell proliferation and differentiation in many tissues, including the thymus. KGF KO mice have impaired thymopoiesis and peripheral T-cell recovery after allogeneic bone marrow transplant (93). Moreover, KGF administration to mice enhance thymopoiesis and accelerate thymic recovery after irradiation (93, 94). In non-human primates, KGF enhances immune reconstitution after autologous hematopoietic progenitor cell transplantation (95, 96).

More recently, conflicting results made the benefits of KGF less clear. Coles et al. (97) reported on treatment with Palifermin (KGF) in patients having received alemtuzumab, a monoclonal anti-CD52 antibody, which induces profound and sustained T cell lymphopenia. Six months after treatment, individuals having received Palifermin had fewer naïve CD4+ T cells and sjTREC, leading to study discontinuation (97). Furthermore, in HIV-infected patients, Palifermin was not effective in either improving thymic function or rising circulating CD4+ T cells (98). Finally, Palifermin was associated with worse clinical outcomes in patients with acute respiratory distress syndrome (99).

All these results underline the difficulty to export results obtained in animal studies to humans and are to make cautious on KGF use.

## Foxn1- and TEC-Based Approaches

Some studies tested whether TEC stem cell may help to restore thymic function.

In a mouse model, Kim et al. (100) reported that engraftment of young TEC allows thymic growth and increased T cell production. FOXN1-induced TEC from fibroblasts support CD4+ and CD8+ T cells development. Transplantation of such cells allows the formation of a complete thymus containing all the TEC subtypes required for T-cell differentiation (101). Another group recently confirmed the feasibility and relevance of such a strategy (102). Moreover, forced expression of FOXN1 in involuted thymus results in thymic regeneration with increased thymopoiesis and naïve T cell output (103). The structure of the regenerated thymus was very close to young thymus in terms of architecture and gene expression. These results suggests that up-regulation of FOXN1 is sufficient to reverse age-related thymic involution. Finally, recombinant FOXN1 protein fused with cell-penetrating peptides increased the number of TEC and enhanced thymopoiesis after hematopoietic stem cell transplantation in mice (104).

All together, these studies suggest that the FOXN1 axis research is a valuable strategy to reverse thymic involution. To date, there are no evaluation of FOXN1 expression during CKD.

## Microbiota

Microbiota interferes with the immune system lifelong and its dysregulation results in inflammation (105). After great variations during the neonatal and early life periods, more subtle changes occur in microbiota until middle age before final stabilisation (106). Nevertheless, age-related changes in intestinal functions, inflammation, and co-morbidities may contribute to dysbiosis (107).

Whether microbiota interferes with immune senescence is challenging because the relative part of microbiota and health status are difficult to isolate. Moreover, even when dysbiosis may favour inflammation, inflammation may also promote dysbiosis asking the question of which came first the chicken or the egg? Indeed, chronic inflammation is a potent driver of increased gut permeability and microbial dysbiosis. For instance, age-induced dysbiosis is reduced in TNF KO mice as compared with wild type (108). Moreover, some cytokines decrease expression of tight junction proteins favours gut permeability, bacterial translocation, and systemic inflammation (109).

There are scarce but convincing data suggesting that aged microbiota contributes to drive immune senescence. Young germ-free (GF) mice raised with aged mice exhibit an inflammatory profile characterised by elevated inflammatory cytokines and macrophage activation (108). This effect was not observed when young GF mice were co-housed with young mice (108). Fransen et al. (109) reported on the transfer of gut microbiota from conventional old mice to young GF mice. T cell activation occurs in young GF mice after transfer of microbiota. Inflammation was related to higher levels of Proteobacteria and lower levels of Akkermansia in old CV mice. Once again, these alterations in immune status were not observed after transfer of microbiota from young conventional mice.

Short-chain fatty acid levels decreased in elderly. Yet, SCFA lead to increase Treg cell differentiation (110). SCFA supplementation, namely butyrate, suppresses arthritis in mice by a Breg-dependent mechanism (111). Precisely, Butyrate increases the levels of 5-HIAA (5-hydroxyindole-3-acetic acid) which activates the ary-hydrocarbon receptor, a transcriptional marker for Breg function (111).

Administration of high dose probiotics in elderly subjects enhanced CD8+CD25+ T cells and NK cells while low dose increased CD4+CD25+ and B lymphocytes (112). A more recent study reported that a probiotic mixture increased naive and regulatory T cells and decreased memory T cells (113).

Finally, best evidence of interactions between microbiota and immune senescence come from studies reporting better vaccine responses against influenza after treatment with pre- or probiotics. Akatsu et al. (114) performed a randomised study in elderly receiving enteral tube feeding. Patients received either a placebo or Bifidobacterium longum BB536. After influenza vaccine, patients having received BB536 exhibited higher levels of anti-H1N1 antibodies. Boge et al. (115) also showed increased response to influenza vaccination in elderly following prolonged administration of a probiotic. Other recent studies suggest that probiotics and prebiotics are effective to improve seroconversion and seroprotection after influenza vaccines (116, 117).

Dysbiosis is a hallmark of chronic kidney disease (118). Accumulation of uremic toxins in CKD leads to an insuatable excretion of urea, uric acid, and oxalates in the intestinal lumen (119). The enrichment in uremic toxins cause substantial modifications in gut physiology mainly an increased in permeability and in microbiota with an increase in uricase and urease-producing bacteria (120). Proteolytic fermentation leads to the formation of different uremic toxins such as p-cresyl sulphate and indoxyl-sulphate potentially aggravating the uremic status (120). Overgrowth of Bacteroidetes, Firmicutes, Ruminococcaceae and clostridia together with low abundance of Lactobacilli, Prevotellae, and bifidobacterium species depict main characteristics of intestinal microbiota of CKD patients compared to healthy subjects (121). Dysbiosis and increased gut permeability take account for bacterial translocation and inflammation (122). More recently, we reported (123) that the proportion of the inflammatory 14-carbon chain lipid A-LPS was increased in ESRD patients compared to healthy volunteers. Conversely, proportion of anti-inflammatory 18-carbon chain lipid A-LPS was decreased. Moreover, sera with predominance of 14-carbon chain lipid A-LPS induced higher secretion of pro-inflammatory cytokines than those with predominance of 18-carbon chain lipid A-LPS. TLR4 or LPS antagonists decreased LPS-induced cytokine production by monocytes, demonstrating an LPS-specific effect. This suggests that septic inflammation observed in ESRD is at least in part related to a shift toward more inflammatory LPS subtypes from altered microbiota.

Different uremic toxins are generated in the intestine and contribute to inflammation in CKD patients (124). p-Cresol is a product of the bacteria metabolization of the aromatic amino acid tyrosine in the colon. Increased levels of p-Cresol in CKD patients correlate with the expansion of terminally differentiated CD8+ T cells (125). A recent randomised study reported that nutritional intervention based on very low protein diet modifies microbiota toward a potential anti-inflammatory profile and reduces p-Cresyl Sulphate (126). This suggests that dietary interventions may mitigate uremic syndrome and immune ageing through microbiota modulation.

## Kidney Transplantation

Successful kidney transplantation reverses renal failure and increases life expectancy. Contrasting effects of kidney transplantation have been observed on immune senescence.

Our group studied markers of immune senescence before and after kidney transplantation. In patients not having received polyclonal antithymocyte globulins (ATG), both T cell relative telomere length and telomerase activity increased after transplantation whereas they were not modified in ATG-treated patients (127). This suggests that renal function recovery may induce a partial reversion of immunesenescence. Nevertheless, Meijers et al. (128) did not observe such changes in T cell RTL after transplantation.

By contrast, concordant data exist to state that thymic output do not increase in non-ATG treated patients and decrease in those having received ATG (127, 128). *In vitro*, ATG binds to TEC and exerts a complement-independent, dose-dependent cytotoxicity (129). Nevertheless, Preville et al. (130) suggested that ATG could not enter into the thymus. Indeed, the authors observed a dose-dependent T cell depletion in spleen and lymph nodes but not in the thymus. However, these first results were not confirmed in a swine model in which lymphodepletion occurs in the thymus after administration of ATG (131). Alternatively, ATG may decrease lymphoid progenitors (127).

ESRD-associated CD8+ T cell expansion tends to marginally increase after transplantation mainly due to CMV reactivation (132). Nevertheless, inflammation measured by CRP or different proinflammatory cytokines substantially dropped after transplantation (122).

All together, these results suggest that kidney transplantation does not reverse ESRD-associated accelerated thymus involution. Whether this absence of effect is due to fixed immune changes or competitive effects of immunosuppressive drugs is difficult to ascertain.

## Dialysis Procedures

Dialysis procedure itself is a source of inflammation. Bioincompatible membranes, prosthetic vascular accesses, PD solution are potential sources of immune activation.

### Peritoneal Dialysis vs. Haemodialysis

Whether PD results in systemic inflammation is not clear. Some studies reported that longer PD duration results in higher IL-6 concentrations (133, 134), but others did not observe any increase in IL-6 or CRP levels (135). By contrast, a burst in inflammation is well-described during HD procedure (136–138). Expression of TLR2 and TLR4 on monocytes from patients on haemodialysis is increased (139) whereas the expression of TLR4 has been reported to be reduced on monocytes in patients with CKD not receiving dialysis (140). Bioincompatible dialyzer induces intermittent activation of monocytes and up-regulation of TLR4. Accordingly, we observed higher inflammatory monocytes counts in patients on HD as compared to those on PD (141).

We also reported higher relative telomerase activity in PD patients (141). Of note, some cytokines released during haemodialysis session, such as IFN-α, may inhibit telomerase activity in hematopoietic cells (142, 143).

Finally, T cell exhaustion was more pronounced in HD patients, especially in those with previous exposure to CMV (141). Different mechanisms may explain this difference. Persistent low-grade inflammation in HD patients may contribute to immune responses to self-antigens and pathological ageing by promoting T cell exhaustion. Alternatively, repeated antigenic stimulation of T cells during haemodialysis session may cause enhanced proliferation and accelerated ageing compared to PD.

### Dialysis Membranes Choice as a Modulator of Inflammation

Bio-incompatible membranes induce sustained activation of innate immunity. During a dialysis session, both neutrophils and monocytes are recruited and activated. After activation, these cells release a number of pro-inflammatory cytokines and complement pathways activators (144). Simultaneously, their phagocytic functions are markedly altered (145, 146). Dialysis membranes also mediate complement activation (147, 148). These phenomena induce persistent pro-inflammatory and pro-coagulant states and partly explain the oxidative burst observed in ESRD patients. Although a direct effect of bio-incompatibility on adaptive immunity is more difficult to demonstrate, inter-connexions between innate and adaptive immunity may explain the consequences of bio-incompatibility on T cell functions (149).

A major challenge to reduce immune activation during dialysis is the development and use of more biocompatible membranes.

Membranes may be modified to reduce oxidative stress. For instance, vitamin E-coded dialyzers reduced indoleamine 2,3-dioxygenase-1 activity and nitric oxide formation (150). Of note, TEC are especially vulnerable to oxidative DNA damage. Thymic stromal deficiency in catalase induces thymic atrophy (151). Treatment with antioxidant can delay the onset of thymus involution (151).

Recently, median cut-off (MCO) membranes characterised by wider pores and more uniformity in pore size were developed. These membranes reduce uremic toxins at a greater degree (152). A randomised study showed that MCO significantly decrease the expression of TNF-α mRNA and IL-6 mRNA in PBMC compared to high-flux dialyzers (153). Polymethyl methacrylate (PMMA) membranes can remove large-weight molecular substances thanks to their adsorptive capacities (154). PMMA membranes seem to be associated with lower pre-dialysis values of IL-6 (155). Contrary to other dialyzers, PMMA membranes are able to clear sCD40 which accumulation in ESRD is associated with unresponsiveness to hepatitis B vaccine (156).

Even when dialysis membrane influence cytokines clearance, complement and coagulation activation, and removal of uremic toxins, a direct impact on immune senescence is not yet proven. However, there are, as described above, several mechanisms linking inflammation and immune ageing. Further studies should examine the effects of different membranes on adaptive immunity, vaccine responses, and clinical outcomes.

### Iron Supplementation

Iron supplementation is widely used in HD (157). Intravenous iron administration induces oxidative stress (158). Iron overload is associated with shorter telomere length in ESRD patients (141). Association between iron overload and telomere length has been reported in different studies (159–161). Reduced telomere length is associated with mortality in dialysis patients (5). Excessive iron load enhances ferroptosis (162), which has an important role in sterile inflammatory conditions such as tissue acute injury, ischemic-reperfusion injury, and neurotoxicity.

## Conclusion

Premature thymic involution and chronic inflammation greatly contribute to increased morbidity and mortality in CKD patients. Mechanisms are likely to be multiple and interlinked. Even when the quest to fountain of youth is a pipe dream, there are many scientific opportunities to prevent or to, at least in part, reverse CKD-related immune senescence. Further studies should precisely define most important pathways driving premature immune ageing in CKD patients and best therapeutic options to control them.

## Author Contributions

DD, ML, and TC wrote the paper. PS and JB corrected the proofs. All authors participate in the works supporting this review.

## Conflict of Interest

The authors declare that the research was conducted in the absence of any commercial or financial relationships that could be construed as a potential conflict of interest.

## Publisher's Note

All claims expressed in this article are solely those of the authors and do not necessarily represent those of their affiliated organizations, or those of the publisher, the editors and the reviewers. Any product that may be evaluated in this article, or claim that may be made by its manufacturer, is not guaranteed or endorsed by the publisher.
